# Data on the characterization of phthalate-degrading bacteria from Asian carp microbiomes and riverine sediments

**DOI:** 10.1016/j.dib.2019.104375

**Published:** 2019-08-09

**Authors:** Steven A. Kolb, Edward J. O'Loughlin, Timothy C. Gsell

**Affiliations:** a Governors State University, Division of Science, Mathematics and Technology, University Park, IL, 60484, USA; b Argonne National Laboratory, Biosciences Division, Argonne, IL, 60439, USA

**Keywords:** Biodegradation, Phthalates, Asian carp, Microbiomes, Bacteria, Isolation

## Abstract

Datasets presented here were employed in the main work “Characterization of phthalate-degrading-bacteria from Asian carp microbiomes and riverine sediments” (Kolb et al., 2019a). The carcinogenic compounds dimethyl phthalate (DMP), diethyl phthalate (DEP), and dibutyl phthalate (DBP) are ubiquitous in the environment due to widespread production and distribution which can be taken up by aquatic organisms. Asian carp species silver (*Hypophthalmichthys molitrix*) and bighead (*Hypophthalmichthys nobilis*) are exposed to phthalates by ingestion and absorption. This article presents data on the characterization of phthalate-degrading bacteria isolated from Asian carp microbiomes and riverine sediments by means of sample collection, enrichment, and isolation. Graphical data presents substrate utilization profiles of consortium SK-1 and *Rhodococcus ruber* derived from the gut microbiome of *H. molitrix*. Additionally, phthalate-degrading microbes were isolated from the gut and scale microbiomes of Asian carp where scanning electron microscopy images show the morphology from samples of final enrichment cultures and isolates. Consortium SK-1 was subjected to amplicon sequencing where community data shows the distribution of taxa while enriched with 500 mg L^−1^ DMP, DEP, and DBP combined. The data presented can provide insights to future research since other phthalate-degrading isolates and consortia can potentially be isolated from the microbiomes of aquatic organisms.

**Table:** Specifications Table

Subject area	*Immunology and Microbiology*
More specific subject area	*Applied Microbiology, Microbiology*
Type of data	*Images, graphs, figures*
How data was acquired	*Sample collection, enrichment, SEM (JEOL JSM-6610LV), UV–Vis Spectrophotometer (Thermo Scientific Spectronic 200), and16S rRNA amplicon sequencing (Illumnia MiSeq Platform)*
Data format	*Raw and Analyzed*
Experimental factors	*Phthalate-degrading bacteria were enriched from sediment and fecal material of Asian carp using mineral salt medium (MSM). Final enrichment cultures and isolates were filtered, dehydrated, and dried prior to SEM. DNA was extracted from consortium SK-1 to identify dominant microbial taxa through 16S rRNA amplicon MiSeq analysis. Inocula were cultured in MSM amended with 1000 mg L*^*−1*^*of mixed phthalates [DMP, DEP, and DBP; each 333.33 mg L*^*−1*^*] prior to growth experiments. During the mid-log phase growth of each inocula (O.D. ≈ 0.6), 2 mL of cell culture was used to inoculate 200 mL of MSM containing phthalates.*
Experimental features	*Two growth experiments were conducted, one system consisting of 1000 mg L*^*−1*^*of either DMP, DEP, or DBP inoculated with Rhodococcus ruber and the other of 500 mg L*^*−1*^*of all 3 phthalates combine, inoculated with either consortium SK-1 or Rhodococcus ruber. Optical density measurements were taken at 600 nm. SEM was used to examine cell morphology during log phase of biodegradation experiments.*
Data source location	*Sediment samples were obtained from Lake Calumet located on the south side of Chicago, IL USA (41.684334, -87.577017). Asian carp (n = 10/species) were captured from the Illinois River in Morris, IL (41.354103, -88.418920).*
Data accessibility	*SK-1 & Rhodococcus OD Raw Data.xlsx*
Related research article	S. A. Kolb, E. J. O'Loughlin & T. C. Gsell, Characterization of phthalate-degrading bacteria from Asian carp microbiomes and riverine sediments, *International Biodeterioration & Biodegradation,* (2019a) IN PRESS [1].

**Table:** 

**Value of the data** • Scanning Electron Microscopy (SEM) data displays morphological characteristics of final enrichment cultures and phthalate-degrading microbes isolated from the gut and scale microbiomes of Asian carp to provide visual representation of cell orientation and evidence of potential interaction of these microbes under high magnification. • Microbial community data of consortium SK-1 enriched from the gut microbiome of *H. molitrix* shows the distribution of genus/family level taxa through enrichment of phthalate-degrading bacteria from the gut microbiomes of other fish species and can act as a reference for other researchers interested in phthalate degradation. • Graphical data of bacterial growth by consortium SK-1 and *Rhodococcus ruber* isolated from SK-1 shows difference in substrate utilization profiles as other researchers may discover that consortia grow faster than isolates. • Image of *Achromobacter aegrifaciens* cells display physical characteristics during the log phase growth when grown on DMP and DEP, may assist in identification or verification that *Achromobacter* species are responsible for this substrate utilization.

## 1 Data

**Fig. 1:**
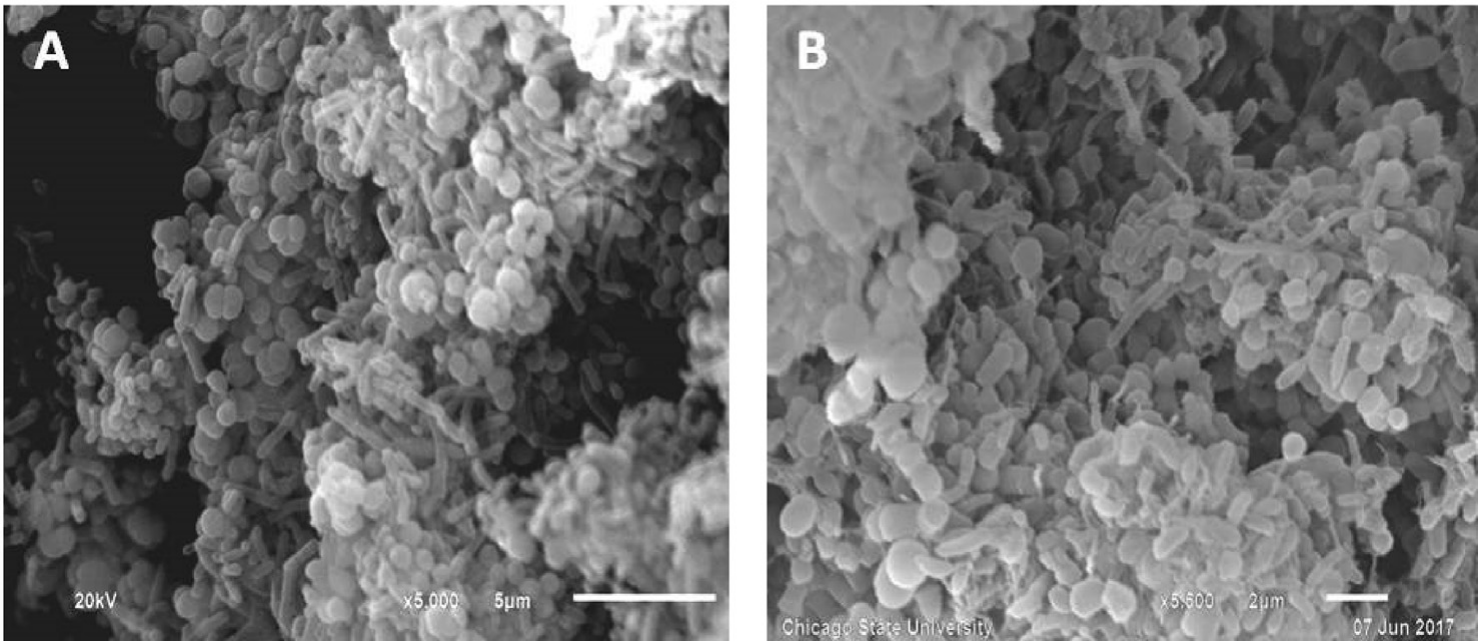
SEM images of A) *Achromobacter* (rod), and *Arthrobacter* (cocci) from a final sediment enrichment culture and B) *Rhodococcus* (cocci), and *Agrobacterium* (rod) from a final *H. molitrix* enrichment culture.

**Fig. 2:**
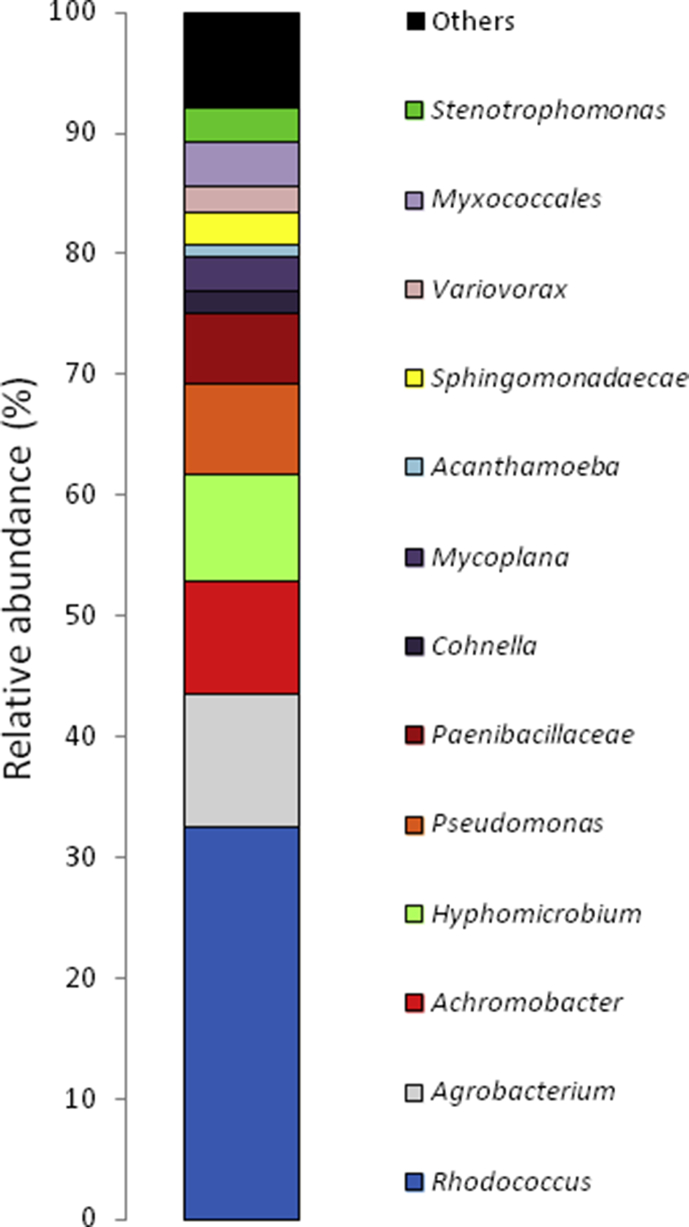
Distribution of genus/family level taxa within consortium SK-1 enriched with mixed phthalates [DMP, DEP, and DBP = 500 mg L^−1^].

**Fig. 3:**
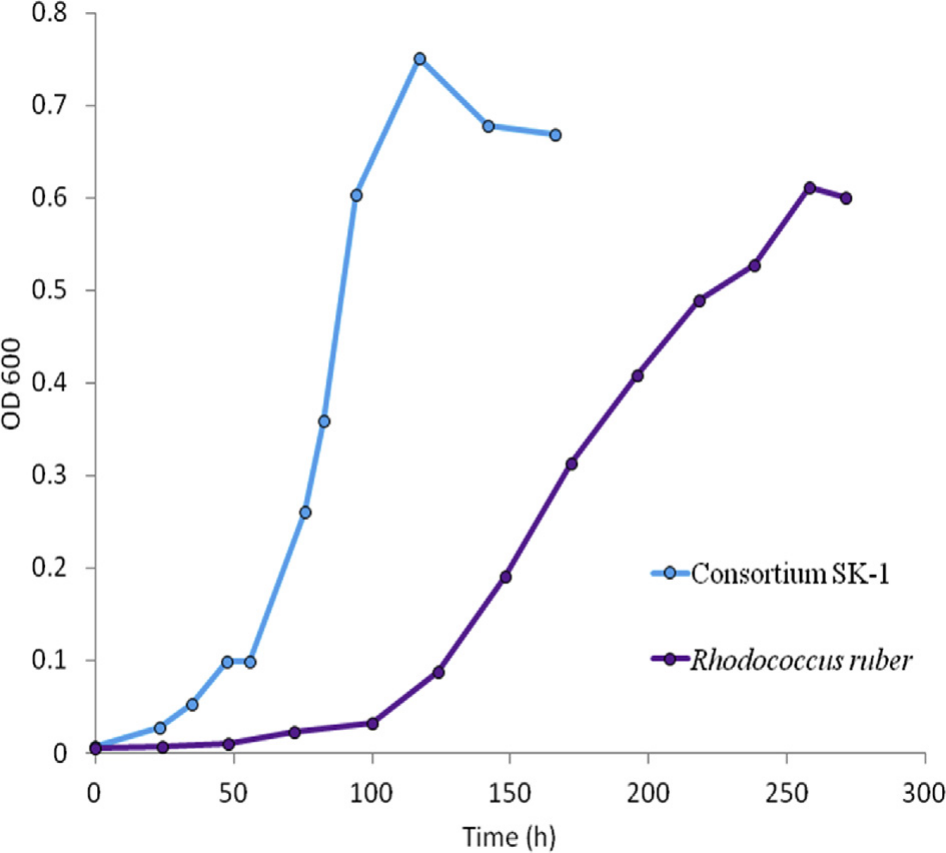
Time course of microbial growth (OD_600_) by consortium SK-1 and *Rhodococcus ruber* on 500 mg L^−1^ phthalate mixture (DMP, DEP, DBP).

**Fig. 4:**
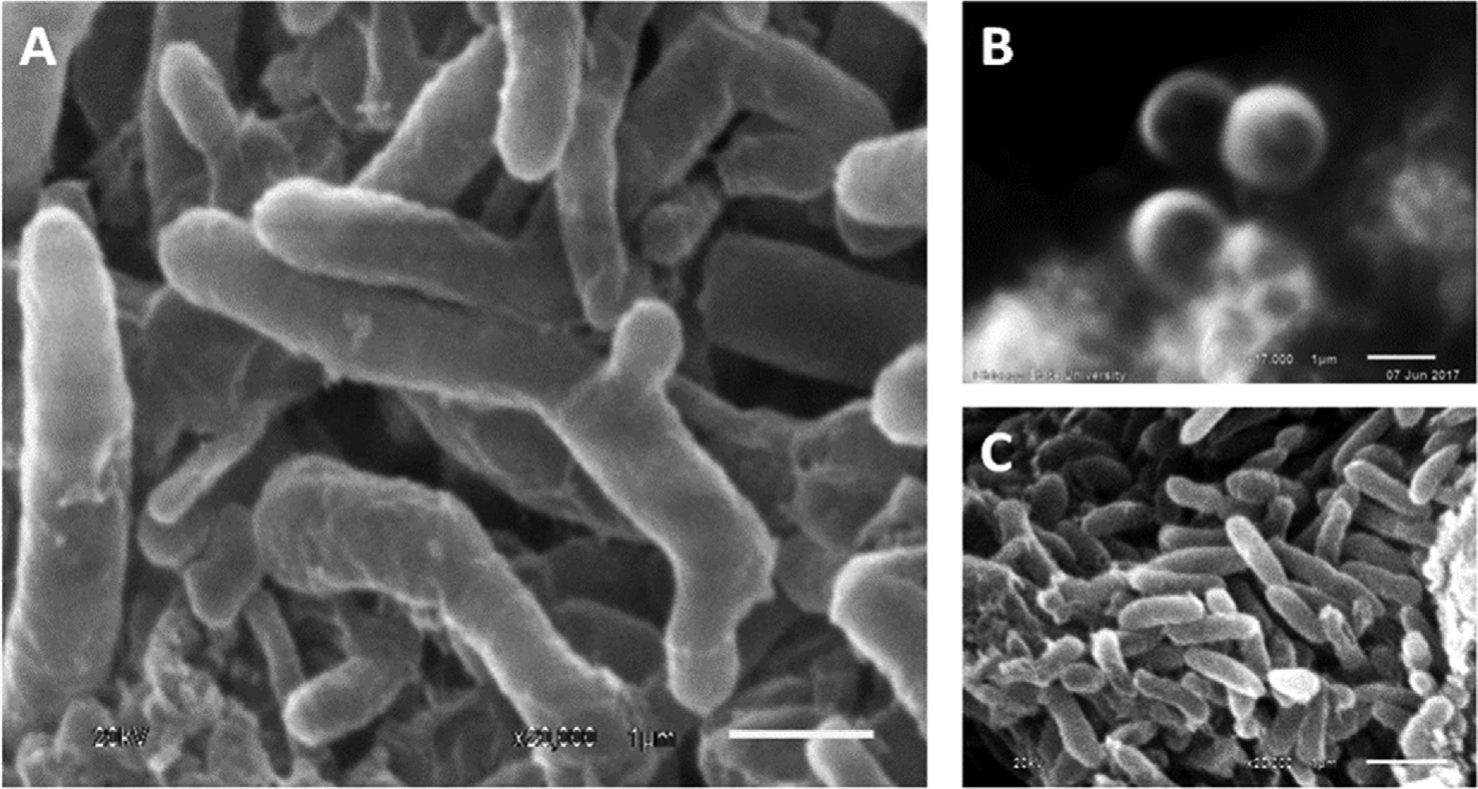
SEM images of strain SK18 (A), *R. ruber* from SK-1 (B), and strain SKTG1 (C) grown on DBP [1000 mg L^−1^].

**Fig. 5:**
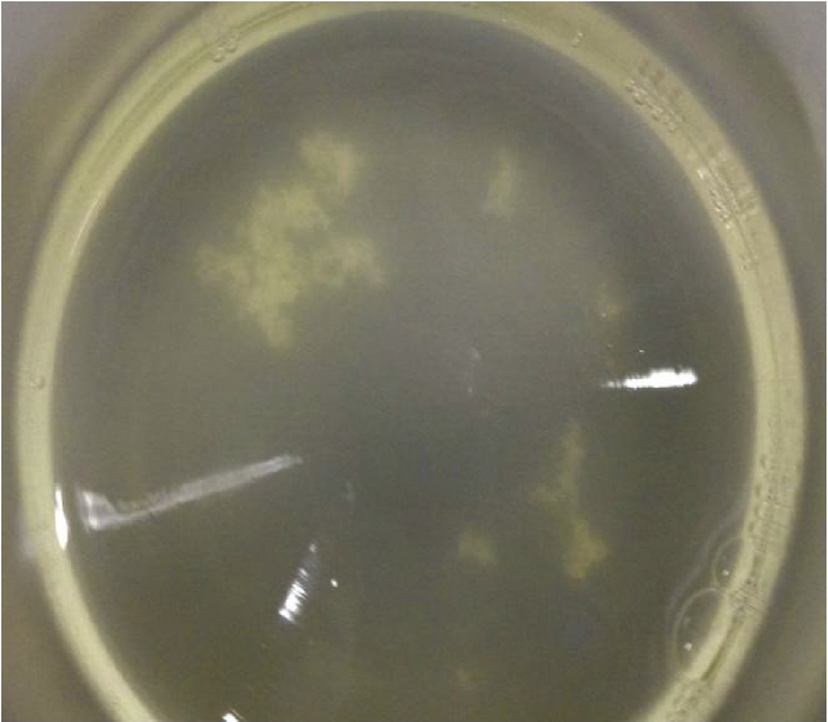
*A. aegrifaciens* cells form clumps in MSM during log phase growth of DMP and DEP; as observed in a similar study with DEHP [5].

**Fig. 6:**
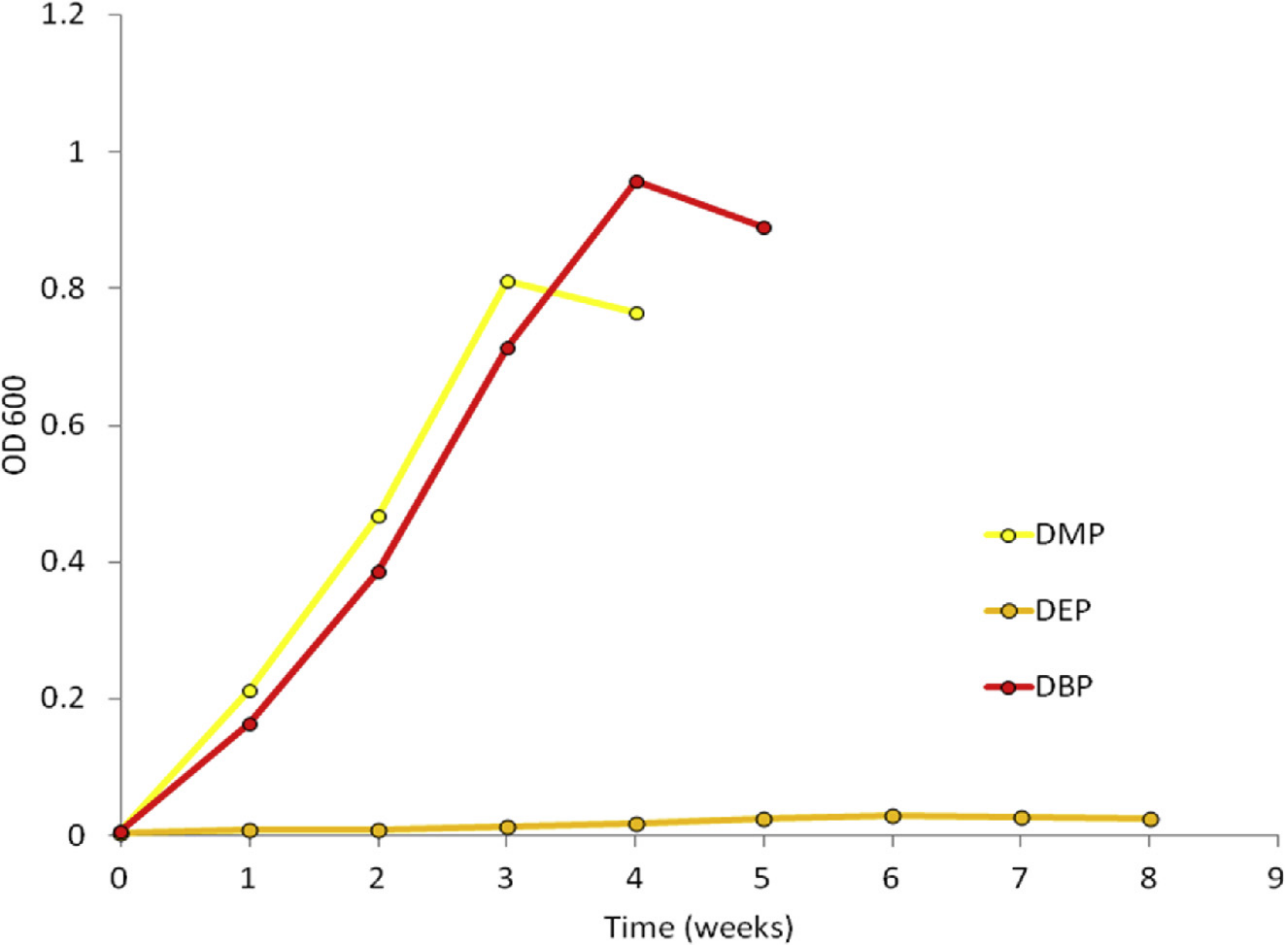
Time course of microbial growth (OD_600_) of DMP and DBP [each 1000 mg L^−1^] by *Rhodococcus ruber*.

The data presented in this article serves to describe the characteristics of phthalate-degrading bacteria from Asian carp microbiomes and riverine sediments [1]. The bacteria isolated in this work were obtained by enriching sediments and Asian carp gut and scale microbiomes with MSM amended with phthalates as previously used by [6] (See Table 1 in [4]. After four serial transfers of sediment or *H. molitrix* gut microbiome*-*inoculated enrichment cultures to fresh MSM (containing 200 → 300 → 400 → 500 mg L^−1^ phthalates), enriched communities were obtained. SEM imaging revealed several cell types during the log phase growth of final enrichment cultures (Fig. 1). A diverse consortium (SK-1) was enriched from the gut microbiome of *H. molitrix* (Fig. 2). At the conclusion of the enrichment process, phthalate-degrading isolates were chosen for further experimentation (see Table 1 in [1]). Isolated from consortium SK-1, *Rhodococcus ruber* exhibited minimal growth on phthalates; however, when SK-1 was intact, rapid growth occurred in cultures that contained a mixture of DMP, DEP, and DBP (500 mg L^−1^) (Fig. 3). Isolated from the gut and scale microbiomes of Asian carp, the cell morphology of *Bacillus subtilis* strain SK18, *Rhodococcus ruber*, and *Pseudomonas putida* strain SKTG1 were examined using SEM (Fig. 4). During the log phase growth of *Achromobacter aegrifaciens* SKTGEO1 on DMP and DEP (see Fig. 4A in [1]), cell clumps formed by auto-aggregation in the MSM (Fig. 5). *Rhodococcus ruber* exhibited maximum biomass production after one month of incubation when amended with DMP and DBP; however, no growth was observed in DEP-amended cultures even after two months of incubation (Fig. 6).

## 2 Experimental design, materials and methods

### 2.1 Sampling locations and collection

Sediment samples were obtained in February of 2017 from Lake Calumet located on the south side of Chicago, IL (41.684334, −87.577017). This location was selected due to the presence of plastic waste, landfill leachates, and unregulated dumping of industrial, municipal, and chemical waste containing the contaminants ethyl-benzene, toluene, and xylene which exceed soil saturation limits [3]. Sediment samples of 0–5 cm depths were gathered by sterile 50 mL tubes fastened to a 3 m pole. Asian carp (n = 10/species) were captured in March of 2017 by the Illinois Department of Natural Resources (IDNR) Aquatic Nuisance Species Program from the Illinois River in Morris, IL (41.354103, −88.418920) as there were numerous industries and landfills downstream. Adult Asian carp were harvested by using gill nets (7.5–10 cm mesh size) and were dissected with sterile equipment to obtain scale tissue and fecal material from the small and large intestines. Sediments and Asian carp samples were immediately frozen after sample collection and stored at −20 °C to minimize changes in the microbial communities.

### 2.2 Isolation of phthalate-degrading bacteria and culture conditions

DMP, DEP, and DBP with >99.0%, >98.0%, and >97.0% purity, respectively, were purchased from TCI America (Portland, Oregon). All other chemicals were high purity (99.0%) and analytical grade. Enrichments for isolation of phthalate-degrading bacteria were prepared in mineral salt medium (MSM) described by [6] (See Table 1 in [4]. The MSM was adjusted to pH 7.0 with 1M NaOH and sterilized by autoclaving for 20 min at 121 °C. Enrichment cultures were prepared in 500 mL Erlenmeyer flasks containing 200 mL of MSM amended with a mixture of DMP, DEP, and DBP (each at 66.66 mg L^−1^, 200 mg L^−1^ total) were inoculated with 5 g (wet weight) of a particular sediment or fecal material sample. The cultures were incubated for 10 days in the dark at 30 °C on a rotary shaker operated at 130 rpm. After incubating for 10 days, 1% by volume of each enrichment was serially transferred to a higher concentration of mixed phthalates (200 → 300 → 400 → 500 mg L^−1^) with a 10 d incubation following each transfer. Samples from the final enrichment were streaked onto MSM Luria-Bertani (LB) agar (MSM amended with 20 g L^−1^ Carolina LB agar) plates supplemented with a mixture of DMP, DEP, and DBP (500 mg L^−1^) and the plates were incubated for 10 days at 30 °C. Isolated colonies (selected on the basis of differences in morphology and coloration) were re-streaked onto MSM LB agar plates with a higher concentration of mixed phthalates (1000 mg L^−1^). Bacterial isolates were further purified by streaking on tryptic soy agar plates and then re-streaked onto MSM LB agar plates with and without phthalates to confirm the ability to grow on phthalates. Isolates that showed increased growth in the presence of mixed phthalates (compared to growth in the absence of phthalates) were selected for growth experiments. Additional enrichments were prepared from the scale biofilm of both Asian carp species. The scale biofilm was swabbed with sterile cotton swabs and transferred onto MSM LB agar plates containing mixed phthalates (200 mg L^−1^) and selected colonies were serially transferred to plates with progressively higher concentrations of mixed phthalates (200 → 300 → 400 → 500 → 1000 mg L^−1^). Isolates that exhibited increased growth relative to increased phthalate concentration were then tested for their ability to grow in liquid MSM for growth experiments and further studied in Kolb et al. (2019).

### 2.3 Community analysis of consortium SK-1

DNA was extracted from consortium SK-1 (MO BIO UltraClean® Microbial DNA Isolation Kit). This was used to identify dominant microbial taxa through 16S rRNA amplicon (151 × 151 bp) MiSeq analysis at the Argonne National Laboratory Sequencing Center (Lemont, IL). The adapter sequences and primers used for the amplification of the V4 hypervariable region of the 16S rRNA gene are described by [2]. To examine microbial communities in consortium SK-1, the relative abundance was calculated from individual taxa at the genus level generated by the 16S rRNA amplicon analysis.

### 2.4 Growth experiments

Consortium SK-1 was tested for its ability to grow on DMP, DEP, and DBP combined as sole carbon sources. Isolated from SK-1, *Rhodococcus ruber* was tested for its ability to grow on each DMP, DEP, and DBP as sole carbon sources individually and combined. Inocula were cultured in MSM amended with 1000 mg L^−1^ of mixed phthalates [DMP, DEP, and DBP; each 333.33 mg L^−1^] prior to growth experiments. During the mid-log phase growth of each inocula (O.D. ≈ 0.6), 2 mL of cell culture was used to inoculate 200 mL of MSM containing 1000 mg L^−1^ of either DMP, DEP, or DBP and a system containing 500 mg L^−1^ of DMP, DEP, and DBP combine. Incubation conditions were the same as described previously. Samples were collected over time to monitor for growth and substrate utilization. In each sampling event: 3 mL of sample was removed for optical density measurements at 600 nm (OD_600_) using a UV–Vis spectrophotometer. Control flasks containing un-inoculated MSM amended with phthalates were also maintained under the same conditions.

### 2.5 Scanning electron microscopy imaging

The cell morphology of enrichments and isolates were examined by extraction of cells from the MSM, which were filtered out of the culture medium at Chicago State University's Core Facility. Cells were centrifuged at 6000×*g* and fixed in 3% glutaraldehyde and 0.1M phosphate buffer for 15 minutes. The cell pellet was dehydrated in a series of aqueous ethanol solutions (30% → 50% → 70% → 95% → 100% ethanol) and dried in a BAL-TEC CPD030 critical point dryer. The cells were then mounted on aluminum stubs with conducting carbon tape. To reduce charging, samples were sputter coated with gold using a Denton Desk II sputter coater. Images were collected using a JEOL JSM-6610LV Scanning Electron Microscope with accelerating voltage of 20 kV.
